# Lung Fibroblasts from Patients with Idiopathic Pulmonary Fibrosis Exhibit Genome-Wide Differences in DNA Methylation Compared to Fibroblasts from Nonfibrotic Lung

**DOI:** 10.1371/journal.pone.0107055

**Published:** 2014-09-12

**Authors:** Steven K. Huang, Anne M. Scruggs, Richard C. McEachin, Eric S. White, Marc Peters-Golden

**Affiliations:** 1 Division of Pulmonary and Critical Care Medicine in the Department of Internal Medicine, University of Michigan, Ann Arbor, Michigan, United States of America; 2 Department of Computational Medicine and Bioinformatics, University of Michigan, Ann Arbor, Michigan, United States of America; Mayo Clinic College of Medicine, United States of America

## Abstract

Excessive fibroproliferation is a central hallmark of idiopathic pulmonary fibrosis (IPF), a chronic, progressive disorder that results in impaired gas exchange and respiratory failure. Fibroblasts are the key effector cells in IPF, and aberrant expression of multiple genes contributes to their excessive fibroproliferative phenotype. DNA methylation changes are critical to the development of many diseases, but the DNA methylome of IPF fibroblasts has never been characterized. Here, we utilized the HumanMethylation 27 array, which assays the DNA methylation level of 27,568 CpG sites across the genome, to compare the DNA methylation patterns of IPF fibroblasts (n = 6) with those of nonfibrotic patient controls (n = 3) and commercially available normal lung fibroblast cell lines (n = 3). We found that multiple CpG sites across the genome are differentially methylated (as defined by P value less than 0.05 and fold change greater than 2) in IPF fibroblasts compared to fibroblasts from nonfibrotic controls. These methylation differences occurred both in genes recognized to be important in fibroproliferation and extracellular matrix generation, as well as in genes not previously recognized to participate in those processes (including organ morphogenesis and potassium ion channels). We used bisulfite sequencing to independently verify DNA methylation differences in 3 genes (*CDKN2B*, *CARD10*, and *MGMT*); these methylation changes corresponded with differences in gene expression at the mRNA and protein level. These differences in DNA methylation were stable throughout multiple cell passages. DNA methylation differences may thus help to explain a proportion of the differences in gene expression previously observed in studies of IPF fibroblasts. Moreover, significant variability in DNA methylation was observed among individual IPF cell lines, suggesting that differences in DNA methylation may contribute to fibroblast heterogeneity among patients with IPF. These results demonstrate that IPF fibroblasts exhibit global differences in DNA methylation that may contribute to the excessive fibroproliferation associated with this disease.

## Introduction

Idiopathic pulmonary fibrosis (IPF) is a devastating and progressive scarring disease of the lung of unknown etiology that results in impaired gas exchange, respiratory failure, and eventually death [1]. Occurring predominantly in older individuals, the median survival of IPF patients is only 3–5 years from the time of diagnosis, with no known effective therapies [1], [2].

The pathologic lesion in IPF is dominated by the presence of increased extracellular matrix proteins and accumulation of fibroblasts, the key effector cell of fibrosis. Fibroblasts from IPF patients have been shown to exhibit multiple differences in phenotype compared to cells isolated from nonfibrotic lung [3]–[7], including increased capacity to proliferate [5], [6], generate extracellular matrix, and resist apoptosis [3], [8]–[10] and antifibrotic signals [4]; these differences were identified in *in vitro* studies, and have consistently been observed to be stable across numerous passages. These phenotypic differences in turn are thought to reflect the differential expression of a variety of genes [3], [4], [7], [8], [11]–[14]. However, the mechanisms that account for such gene expression differences are unknown.

DNA methylation is a critical means of gene regulation and aberrant DNA methylation is important in the development of many diseases; however, explorations into its potential role in IPF have been limited. Studies utilizing whole tissue have identified global methylation differences between the lungs of IPF patients and individuals without fibrotic lung disease [15], [16]. However, since the lung contains up to forty distinct cell types and since such studies cannot localize these methylation differences to individual cell types, the pathobiologic significance of such methylation differences remain uncertain. Regulatory regions of select genes have been shown to be differentially methylated in IPF fibroblasts [17]–[19], but a global analysis of methylation differences in IPF fibroblasts has not been reported.

Here, we compare the global DNA methylation profile between IPF fibroblasts and two groups of controls, and identify differences in DNA methylation at many loci. Selected methylation differences were independently validated by bisulfite sequencing, and were found to functionally contribute to altered gene expression. These methylation differences were found in genes enriched in specific gene ontology classes – some with recognized relevance to fibrosis and others that might be considered unexpected based on our current understanding of fibrogenesis. Our data thus provide the opportunity to identify novel genes that are differentially methylated and differentially expressed, which may contribute to the pathogenesis of IPF.

## Methods

### Cell culture

IPF fibroblasts were outgrown from lung tissue obtained by surgical lung biopsy of patients with IPF, as previously described [20]. All biopsy specimens were confirmed histologically to show the characteristic histopathologic pattern termed usual interstitial pneumonia. Control fibroblasts were cultured from histologically normal regions of lung from age-matched patients undergoing resection for lung nodules. Because these nodules often proved to be cancer, we also used as an additional control commercially available primary fibroblast lines (CCL204, CCL190, and CCL210, all from American Type Culture Collection, Manassas, VA) grown from the lungs of adults with no preexisting lung disease, to rule out the possibility that any observed methylation differences might be the consequence of a “field effect” of cancer [21], [22]. Patient-related data are shown in Table 1. Independent comparisons were made between IPF and these two nonfibrotic control groups. All fibroblasts were cultured in DMEM (Invitrogen, Carlsbad, California) supplemented with 10% fetal bovine serum (Hyclone, Logan, Utah) and 100 U/ml pencillin/streptomycin at 37°C with 5% CO_2_, and examined between passages 4–6.

**Table 1:** Demographic data of patient-derived cell lines.

Cell line	Pathologic diagnosis	Age*	Gender	Smoking (pk-yrs)	FVC (% pred)*	DLCO (% pred)*	TLC (% pred)*	Honeycomb on CT
**Nonfibrotic controls**								
NF-1	Hamartoma	59	M	10	119	102	NA	No
NF-2	Lung CA	58	M	50	98	NA	NA	No
NF-3	Lung CA	53	M	20	88	NA	NA	No
**Commercial nonfibrotic cell lines**								
CCL190	Nml lung, died of sarcoma	18	M					
CCL204	Nml lung, died of astrocytoma	35	M					
CCL210	Nml lung, died of head trauma	20	F					
**IPF**								
IPF-1	UIP	65	F	67.5	72	57	73	No
IPF-2	UIP	44	F	0	52	39	57	No
IPF-3	UIP	67	F	20	62	37	71	Yes
IPF-4	UIP	46	F	NA	39	29	49	No
IPF-5	UIP	70	M	25	48	35	56	No
IPF-6	UIP	Not available; de-identified lung from IPF patient obtained at time of transplant						

*prior to biopsy; DLCO (diffusing capacity for carbon monoxide); TLC (total lung capacity); FVC (forced vital capacity).

### DNA methylation analysis

To analyze global DNA methylation patterns, 1 µg of genomic DNA was subjected to bisulfite conversion using the EZ DNA Methylation Kit from Zymo Research (Irvine, California). Bisulfite-converted DNA was analyzed for methylation at 27,578 CpG sites using the Illumina (San Diego, California) HumanMethylation27 BeadChip Array according to the manufacturer’s protocol. Signal intensity from methylated and unmethylated probes for all sites was scanned on the Illumina BeadArray Reader, and preprocessed using Illumina GenomeStudio software. The methylation status of individual CpG sites was verified by pyrosequencing. Bisulfite-modified DNA was amplified by PCR using biotin-labeled primers specific for the *CDKN2B*, *MGMT*, and *CARD10* promoters. The primer sequences for *CDKN2B* and *CARD10* are shown in Table S1; primers for *MGMT* (assays ASY514FS and FS1) were obtained from EpigenDx (Hopkinton, Massachusetts). The biotinylated PCR product was then bound to beads, washed through the Vacuum Prep Tool (Qiagen, Valencia, California), and mixed with sequence-specific primers before analysis on the Pyrosequencer (Qiagen).

### RT-PCR and immunoblot

RNA was isolated from cells using Trizol (Invitrogen), and quantitative mRNA levels of CDNK2B were assayed using the StepOne Real-time PCR System (Applied Biosystems, Carlsbad, California), with primers from Applied Biosystems. Quantitative values were obtained relative to human β-actin which was used as endogenous control, with β-actin primer and probe sequences previously reported [23]. Cell lysates were collected in lysis buffer (PBS containing 1% Nonidet P-40, 0.5% sodium deoxycholate, 0.1% SDS, 2 mM orthovanadate, and protease cocktail inhibitor), resolved by SDS-PAGE, transferred to nitrocellulose membranes, and immunoblotted using antibodies to CDKN2B (1∶1000, Thermo Scientific, Rockford, Illinois), MGMT (1∶1000, Cell Signaling, Beverly, Massachusetts), CARD10 (1∶1000, Abcam, Cambridge, Massachusetts), and α-tubulin (1∶1000, Sigma-Aldrich, St. Louis, Missouri). Bound primary antibodies were visualized with appropriate secondary antibody conjugated to horseradish peroxidase and developed with enhanced chemiluminescence reagent (GE Healthcare, Piscataway, New Jersey). Densitometric analysis was performed on the visualized bands using Image J Software (NIH, Bethesda, Maryland).

### Cell proliferation

Cells were plated at 2×10^3^ cells/well in 96-well plates and treated with either control siRNA or siRNA targeting CDKN2B (ON-TARGETplus SMARTpool, Thermo Scientific). After 72 h, cell proliferation was assayed by the CyQuant assay (Life Technologies) per manufacturer’s protocol.

### Data analysis

All methylation array data have been deposited in the National Center for Biotechnology Information Gene Expression Omnibus (GEO) database under accession number GSE56074. For the HumanMethylation27 array data, signal intensities were corrected for red/green color balance, adjusted for background signal, and normalized across the set of arrays. M-values were calculated as the log2 ratio of the intensities of the methylated probe versus unmethylated probe as described by Du et al [24]. M-values were chosen over beta-values for differential methylation analysis because of their reported superior performance [24]. M-values were compared between IPF and both nonfibrotic control groups and statistically significant differences were identified using the limma algorithm in the Bioconductor R suite (http://www.bioconductor.org) (22). Significant differences in M values were defined using thresholds of p-value less than 0.05 and a fold change of greater than 2; this “fold change ranking with a non-stringent p-value cutoff” approach has been validated by other microarray studies [25]–[27] and was shown to perform well with M-values [24]. CpG loci with known single nucleotide polymorphism (SNP) annotations were identified from dbSNP 132, as suggested at http://rforge.net/IMA/. Gene enrichment analysis was performed using ConceptGen (http://conceptgen.ncibi.org) with Q<0.05 defined as statistically significant. Network analysis of gene concepts was performed using STITCH (http://stitch.embl.de/). All other data were analyzed on GraphPad Prism 5.0 (GraphPad Prism Software, San Diego, CA) using ANOVA or Student’s t-test, as appropriate, with a p<0.05 defined as statistically significant. Data are expressed as mean ± SEM.

### Ethics statement

All patients provided written informed consent and protocols were approved by the University of Michigan Institutional Review Board (HUM00023700).

## Results

### IPF fibroblasts exhibit global differences in DNA methylation compared to nonfibrotic control cells

**Figure 1:**
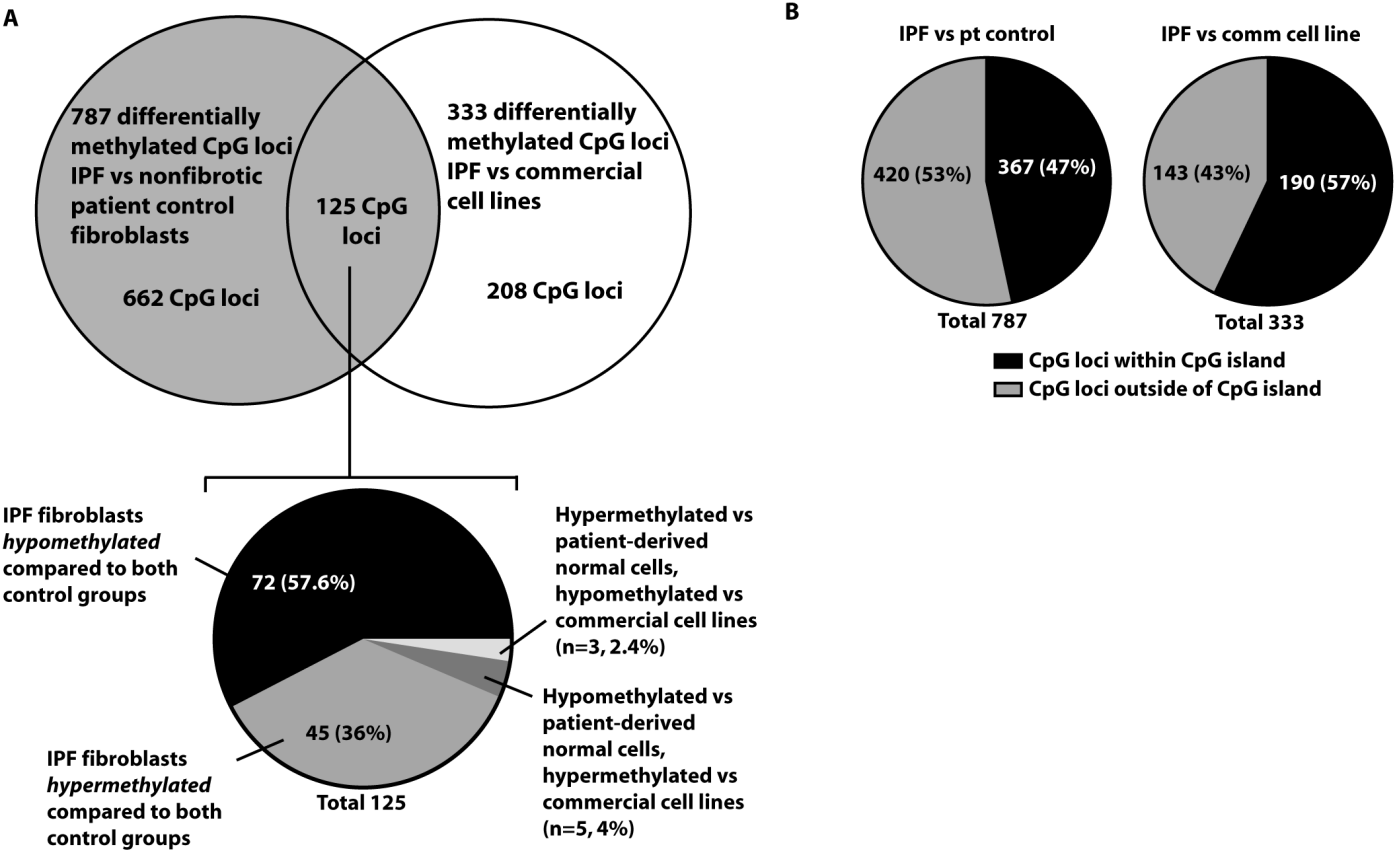
Methylation differences between IPF fibroblasts and two groups of nonfibrotic control cells. Levels of DNA methylation were analyzed using the HumanMethylation27 array in 6 IPF fibroblast lines, 3 patient-derived nonfibrotic controls, and 3 commercially available nonfibrotic cell lines (CCL190, CCL204, and CCL210). A) The number of differentially methylated CpG loci between IPF and patient-derived controls, between IPF and commercial cell line controls, and the overlap of these differences are shown. B) Fraction of differentially methylated CpG loci that are within and outside of CpG islands.

Fibroblasts from the lungs of 6 IPF patients and 3 age-matched control patients without fibrotic lung disease were cultured and their DNA methylation profiles were analyzed on the HumanMethylation27 Bead-Chip array. The demographic characteristics of the patients are shown in Table 1. A second control group consisted of the commercial cell lines CCL190, CCL204, and CCL210, which are primary fibroblast lines grown from the lungs of individuals without lung cancer or fibrotic lung disease. Using the significance threshold criteria described in Methods, we identified 787 CpG loci that were differentially (either increased or decreased) methylated (listed in GEO database accession GSE56074) in IPF versus patient-derived control cells. The top 50 differentially methylated CpG loci, based on p-value and sorted by fold change, are shown in Table S2. When IPF cells were compared to the three commercial control cell lines, using the same selection criteria, 333 CpG loci were differentially methylated (GEO accession GSE56074). The top 50 CpG loci, based on p-value and sorted by fold change, are shown in Table S3. Of these two groups of differentially methylated CpG loci, 125 were identified to be shared (listed in Table S4), of which 72 were identified as concurrently hypomethylated and 45 were identified as concurrently hypermethylated in IPF cells when compared to either patient-derived or commercial cell line control cells (Figure 1A). Table 2 lists those differentially methylated CpG loci, and their associated genes, that were found in common in the lists of 50 most-differentially methylated CpG loci between the two control groups.

**Table 2:** Differentially Methylated CpG Sites In Common Among the Top 50 Identified When IPF Cells Were Compared to Both Control Groups.

				IPF vs patient-derived lines		IPF vs comm cell lines	
Gene ID	Gene Symbol	Gene Name	CpG MapInfo#	log FC*	p value	log FC*	p value
1030	CDKN2B	cyclin-dependent kinase inhibitor 2B(p15, inhibits CDK4)	9:21995769	2.60	1.66E-05	2.19	1.19E-04
29775	CARD10	caspase recruitment domain family,member 10	22:36244805	1.62	6.58E-05	1.29	6.73E-04
7419	VDAC3	voltage-dependent anion channel 3	8:42367859	−2.11	1.06E-05	−1.61	2.36E-04
5724	PTAFR	platelet-activating factor receptor	1:28376307	−1.18	6.63E-04	−1.25	3.89E-04
6506	SLC1A2	solute carrier family 1(glial high affinity glutamate transporter),member 2	11:35397101	1.83	1.39E-04	1.43	1.48E-03
79041	TMEM38A	transmembrane protein 38A	19:16632394$	−1.39	8.73E-05	−1.00	2.03E-03
60529	ALX4	aristaless-like homeobox 4	11:44284148	2.43	5.76E-04	2.07	2.30E-03
55867	SLC22A11	solute carrier family 22(organic anion/cation transporter),member 11	11:64079466$	−1.86	2.46E-05	−1.35	7.61E-04
54808	DYM	dymeclin	18:45241530	1.49	4.73E-05	1.66	1.28E-05
353219	KAAG1	kidney associated antigen 1	6:24465699	2.33	2.86E-04	1.90	1.79E-03
339366	ADAMTSL5	ADAMTS-like 5	19:1463314	1.83	7.68E-04	1.62	2.27E-03
200172	SLFNL1	schlafen-like 1	1:41259495	1.18	1.88E-04	1.28	7.56E-05

# NCBI Build 36.1;

*log FC = log fold change in methylation of IPF fibroblasts vs nonfibrotic fibroblasts;

$ CpG site annotated in dpSNP v132 database for known single nucleotide polymorphism.

Using the database of SNPs dbSNP 132, 3,690 of 27,578 CpG loci (13.4%) on the HumanMethylation27 array are annotated for known SNPs. Among the 787 CpG loci that were differentially methylated between IPF and patient-derived control cells, 116 (14.7%) were annotated for SNPs. Among the 333 CpG loci that were differentially methylated between IPF and commercial cell lines, 46 (13.8%) were annotated for SNPs. Since the proportion of annotated SNPs in the dataset is similar to the proportion of SNPs annotated over the entire array, the data do not suggest that SNPs represent a bias in the identification of differentially methylated CpG sites (Chi-Square p-values 0.29 and 0.82 for the two different control comparisons). CpG sites that have annotated SNPs are indicated in Tables 2, S2, S3, and S4.

The HumanMethylation27 array was designed to be biased towards analyzing CpG sites that are near gene promoters and located within CpG islands (72.5% of probes). Despite this bias, the majority (53%) of differentially methylated CpG fell outside of CpG islands (Figure 1B).

**Figure 2:**
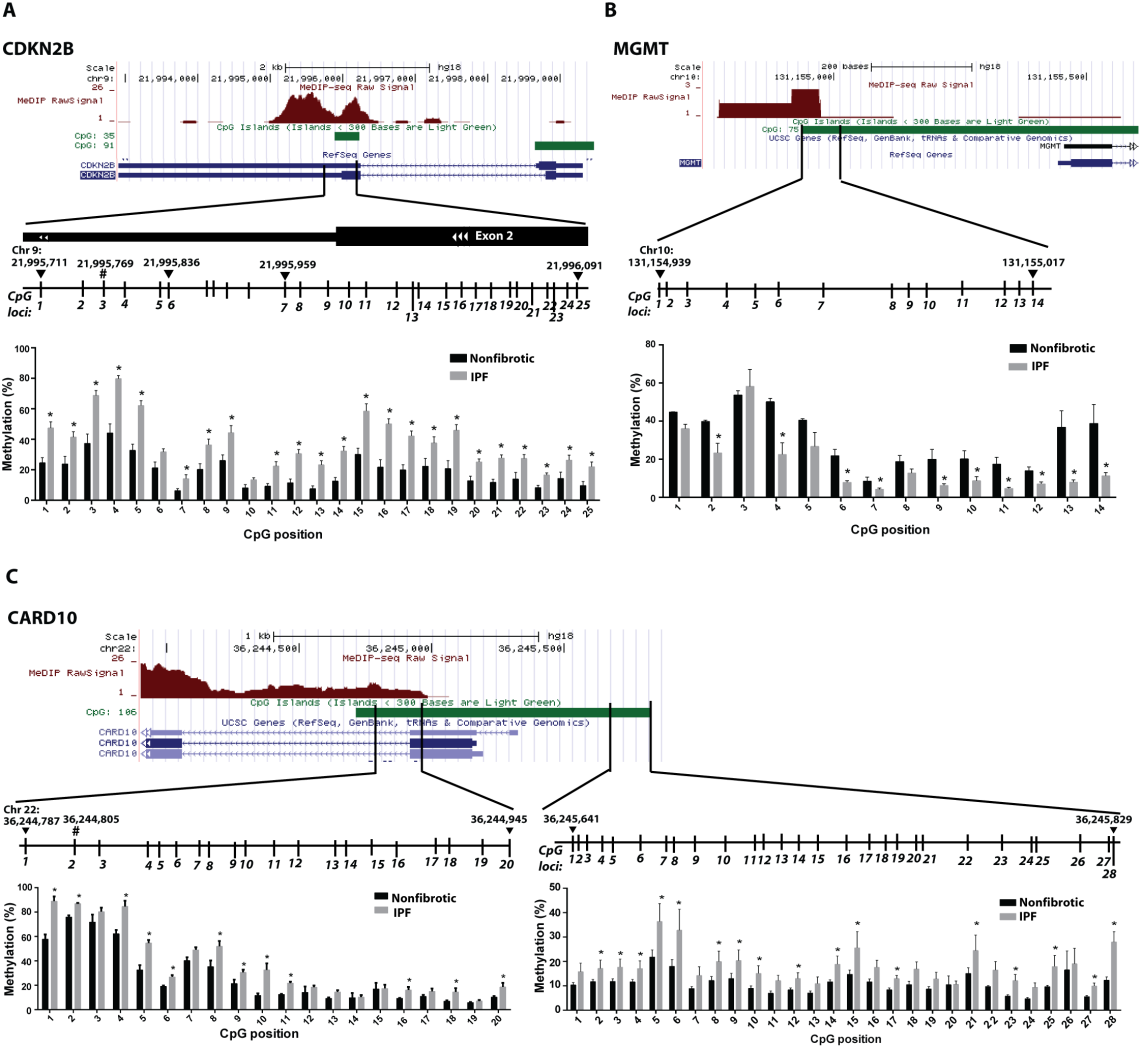
Methylation levels of *CDKN2B*, *CARD10*, and *MGMT* by bisulfite sequencing in IPF and nonfibrotic control fibroblasts. The DNA methylation levels of various CpG sites within the *CDKN2B* (A), *CARD10* (B), and *MGMT* (C) genes were analyzed by bisulfite pyrosequencing in fibroblasts from IPF (n = 6) and nonfibrotic control patients (n = 3). The hashtag indicates the CpG site that was assayed and identified to be differentially methylated by the array. Illustrated are the location of the CpG sites analyzed (based on NCBI Build 36.1) and position relative to the gene location, theoretical CpG islands, and MeDIP-Seq data from UCSC Genome Browser. *P<0.05.

To independently verify differences in DNA methylation between IPF and control cells, we performed bisulfite sequencing of three specific genes – *CDKN2B*, *MGMT*, and *CARD10*– that were identified by the array as differentially methylated in IPF fibroblasts. *CDKN2B* and *CARD10* were chosen for validation because they were the top two genes identified by the array as being hypermethylated in IPF cells compared to both control groups (Table 2). *MGMT* was chosen because it was a hypomethylated gene in IPF cells that we had previously shown was hypermethylated following treatment with an antifibrotic mediator, prostaglandin E_2_ [28], whose endogenous biosynthesis is diminished in IPF [14]. Methylation analysis of 25 CpG sites within the *CDKN2B* gene, which included the site interrogated by the array, revealed that IPF fibroblasts were hypermethylated at nearly every CpG site sequenced (Figure 2A). In contrast, the *MGMT* promoter was hypomethylated at 10 of 14 CpG sites analyzed in IPF fibroblasts compared to nonfibrotic control cells, which is consistent with the array results (Figure 2B). The differential methylation of *CDKN2B* and *MGMT* in IPF fibroblasts was largely localized to CpG islands, and in regions where reference sequencing showed other cell types to also be differentially methylated (as indicated by MeDIP-Seq reference data [29]). Bisulfite sequencing of the *CARD10* promoter revealed that like *CDKN2B*, it too was hypermethylated in IPF fibroblasts (Figure 2C). The identified hypermethylated regions include the CpG site that was assayed by the array, as well as regions up- and downstream of the transcription start site. Interestingly, hypermethylation was identified in two separate regions, one in which MeDIP-Seq signaling was present, and one in which MeDIP-Seq signaling was absent in reference data [29].

### Aberrant methylation of CDKN2B, CARD10, and MGMT in IPF fibroblasts is associated with altered gene expression and increased fibroblast proliferation

**Figure 3:**
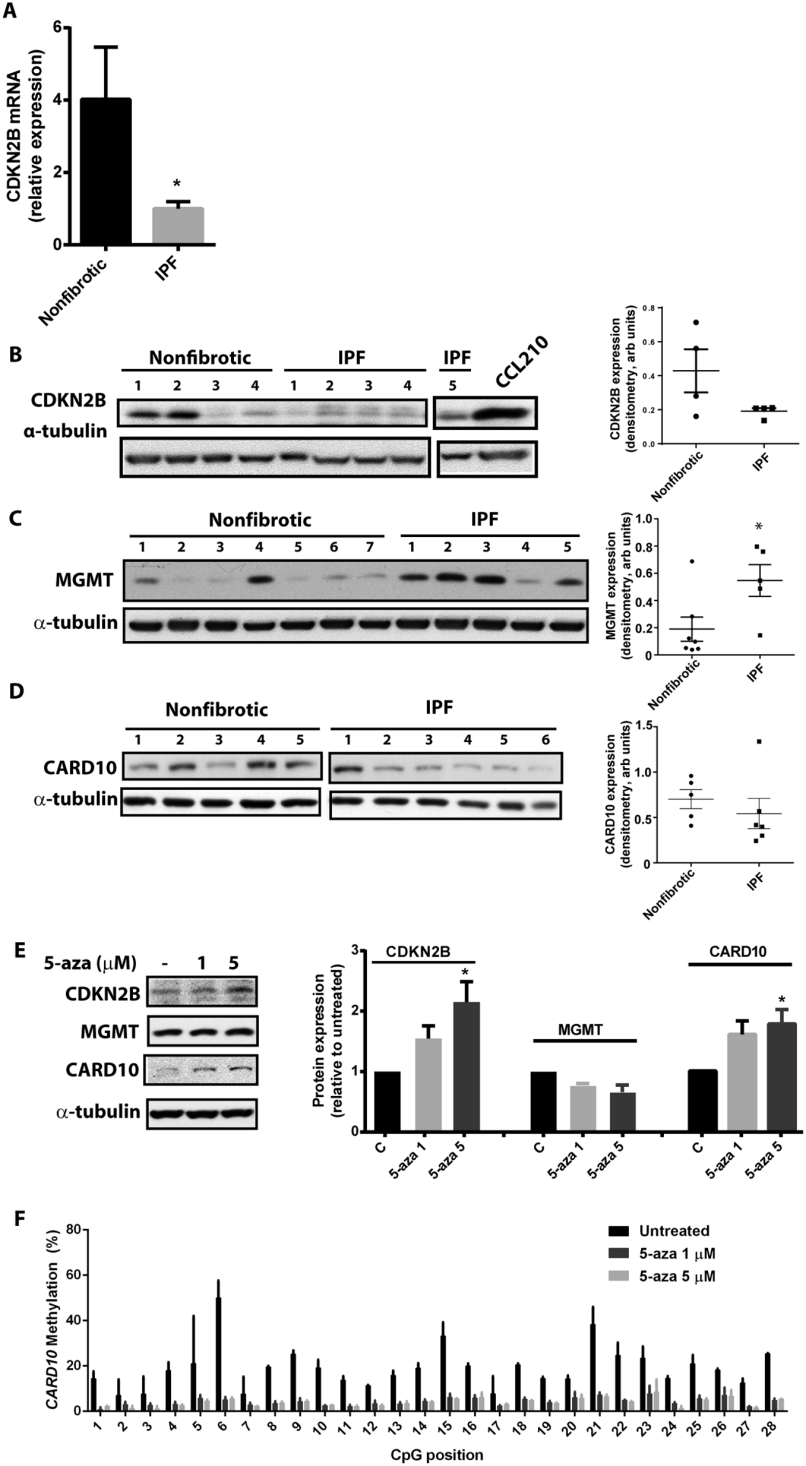
Expression of CDKN2B, CARD10, and MGMT in IPF and nonfibrotic control fibroblasts. Expression of (A) CDKN2B mRNA (n = 3 nonfibrotic, n = 5 IPF), (B) CDKN2B protein, (C) MGMT protein, and (D) CARD10 protein were assayed in IPF and nonfibrotic control fibroblasts. (E) IPF cells were treated with the DNA methylation inhibitor 5-aza-2′-deoxycytidine (5-aza) at the indicated concentrations, and expression of CDKN2B, CARD10, and MGMT were assayed by immunoblot relative to α-tubulin and normalized to untreated control (n = 3). (F) The methylation of the *CARD10* gene promoter was assayed in IPF cells after 72 h treatment with 5-aza or vehicle control (n = 2). *P<0.05.

**Figure 4:**
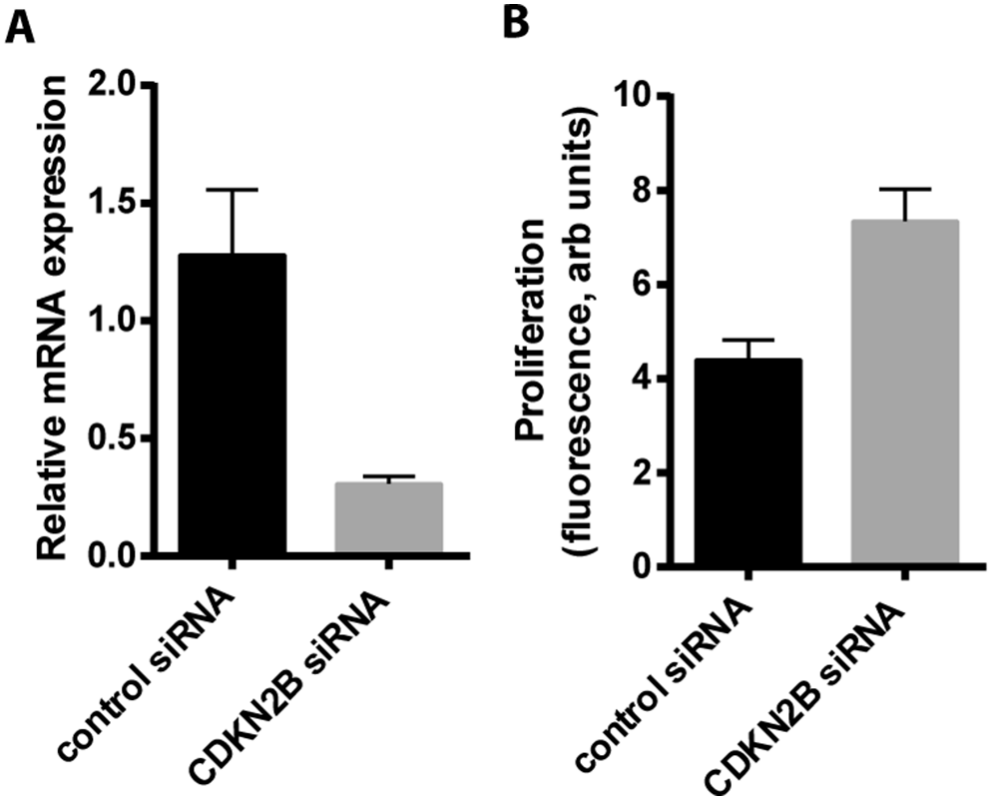
Silencing of CDKN2B and cell proliferation. CCL210 fibroblasts were treated with either control siRNA or siRNA targeted against CDKN2B. A) Levels of CDKN2B mRNA was assayed by RT-PCR (n = 3). B) Cell proliferation was measured by the CyQuant assay. Shown are the mean data from 6 replicates of a representative experiment.

DNA methylation, especially within gene promoters and CpG islands, is traditionally associated with decreased gene expression. To determine the impact of altered DNA methylation on gene expression, we compared the expression of CDKN2B, CARD10, and MGMT mRNA and protein in IPF fibroblasts with those of patient control cells. IPF cells exhibited decreased expression of CDKN2B and CARD10 and increased expression of MGMT compared to nonfibrotic control fibroblasts (Figure 3A–D), concordant with the observations that CDKN2B and CARD10 were hypermethylated and MGMT was hypomethylated in IPF cells. Treating IPF fibroblasts with the DNA methylation inhibitor 5-aza-2′-deoxycytidine resulted in increased expression of CDKN2B and CARD10, but not MGMT (Figure 3E), consistent with the hypothesis that increased methylation down-regulates expression of CKDN2B and CARD10. Treatment with 5-aza-2′-deoxycytidine was associated with a decrease in *CARD10* methylation (Figure 3F), and a more modest decline in the methylation of *CDKN2B* (from 37.9% to 31%, based on the mean of the 28 CpG loci assayed). As expected, expression of MGMT did not change with methylation inhibitors, as MGMT was hypomethylated in IPF cells. CDKN2B is an inhibitor of cyclin-dependent kinases 4 and 6 and is a key regulator of cell cycle arrest [30]; thus, increased methylation followed by decreased CDKN2B expression might be expected to contribute to the reported increase in proliferation observed in IPF fibroblasts [5], [6]. Consistent with this possibility, silencing CDKN2B expression by siRNA in normal fibroblasts resulted in increased cell proliferation (Figure 4).

### Aberrant DNA methylation may account for the differential expression of other genes identified in IPF fibroblasts

Previous studies have shown that many genes are differentially expressed in IPF fibroblasts. We compared our set of genes that were differentially methylated between IPF fibroblasts and patient-derived controls with those genes identified in a study by Lindahl et al (GEO accession GSE40839) as being differentially expressed in IPF cells [31], and identified 52 genes in common between the datasets (q-value 7.23×10^−5^). These genes are shown in Table 3.

**Table 3:** Genes Differentially Methylated and Expressed.

Gene ID	Gene Symbol	Gene name	log FCexpression*	log FC methylation
6372	CXCL6	chemokine (C-X-C motif) ligand 6(granulocyte chemotactic protein 2)	−4.14	−1.22
220	ALDH1A3	aldehyde dehydrogenase 1 family,member A3	−4.1	1.51
5359	PLSCR1	phospholipid scramblase 1	−3.93	1.93
81035	COLEC12	collectin sub-family member 12	−3.76	1.42
6355	CCL8	chemokine (C-C motif) ligand 8	−3.68	−1.86
64761	PARP12	poly (ADP-ribose) polymerase family,member 12	−3.41	1.45
7133	TNFRSF1B	tumor necrosis factor receptor superfamily, member 1B	−3.36	−2.13
57664	PLEKHA4	pleckstrin homology domain containing,family A (phosphoinositide bindingspecific) member 4	−3.19	−1.57
6474	SHOX2	short stature homeobox 2	−2.96	1.93
5493	PPL	periplakin	−2.82	1.18
8644	AKR1C3	aldo-keto reductase family 1, member C3(3-alpha hydroxysteroid dehydrogenase,type II)	−2.7	−1.46
3428	IFI16	interferon, gamma-inducible protein 16	−2.65	−1.44
1513	CTSK	cathepsin K	−2.63	−1.86
7049	TGFBR3	transforming growth factor, beta receptor III	−2.44	2.43
7291	TWIST1	twist homolog 1 (acrocephalosyndactyly 3; Saethre-Chotzen syndrome)(Drosophila)	−2.24	1.04
7424	VEGFC	vascular endothelial growth factor C	−2.19	−1.50
9536	PTGES	prostaglandin E synthase	−2.09	−2.44
2766	GMPR	guanosine monophosphate reductase	−2.07	1.36
5142	PDE4B	phosphodiesterase 4B, cAMP-specific (phosphodiesterase E4 dunce homolog, Drosophila)	−1.8	−1.08
9603	NFE2L3	nuclear factor (erythroid-derived 2)-like 3	−1.5	1.11
1534	CYB561	cytochrome b-561	−1.48	−2.16
23433	RHOQ	ras homolog gene family, member Q	−1.46	1.38
4601	MXI1	MAX interactor 1	−1.43	1.24
5156	PDGFRA	platelet-derived growth factor receptor,alpha polypeptide	−1.41	−1.28
9641	IKBKE	inhibitor of kappa light polypeptide geneenhancer in B-cells, kinase epsilon	−1.23	−1.53
4281	MID1	midline 1 (Opitz/BBB syndrome)	−1.17	−1.29
8462	KLF11	Kruppel-like factor 11	−1.12	−2.12
25960	GPR124	G protein-coupled receptor 124	−1.03	−1.02
6274	S100A3	S100 calcium binding protein A3	−0.999	−1.31
717	C2	complement component 2	−0.987	−1.36
3964	LGALS8	lectin, galactoside-binding, soluble, 8 (galectin 8)	−0.979	−2.54
3460	IFNGR2	interferon gamma receptor 2 (interferon gamma transducer 1)	−0.863	1.34
23268	DNMBP	dynamin binding protein	−0.828	1.15
2260	FGFR1	fibroblast growth factor receptor 1(fms-related tyrosine kinase 2, Pfeiffer syndrome)	−0.813	−1.43
975	CD81	CD81 molecule	−0.733	1.28
11078	TRIOBP	TRIO and F-actin binding protein	−0.684	1.84
9391	CIAO1	cytosolic iron-sulfur protein assembly 1homolog (S. cerevisiae)	0.681	−2.44
3655	ITGA6	integrin, alpha 6	1.02	−1.04
2346	FOLH1	folate hydrolase (prostate-specific membrane antigen) 1	1.1	−1.56
27286	SRPX2	sushi-repeat-containing protein, X-linked 2	1.12	−1.48
23187	PHLDB1	pleckstrin homology-like domain, family B, member 1	1.16	−1.67
3987	LIMS1	LIM and senescent cell antigen-like domains 1	1.35	−1.04
5947	RBP1	retinol binding protein 1, cellular	1.38	1.09
10962	MLLT11	myeloid/lymphoid or mixed-lineageleukemia (trithorax homolog, Drosophila); translocated to, 11	1.58	−1.78
326	AIRE	autoimmune regulator(autoimmune polyendocrinopathy candidiasis ectodermal dystrophy)	1.61	−1.27
8577	TMEFF1	transmembrane protein with EGF-like and two follistatin-like domains 1	1.67	1.43
4162	MCAM	melanoma cell adhesion molecule	2.07	1.34
3918	LAMC2	laminin, gamma 2	2.54	1.74
50615	IL21R	interleukin 21 receptor	2.73	1.60
2246	FGF1	fibroblast growth factor 1 (acidic)	2.79	1.41
55214	LEPREL1	leprecan-like 1	2.82	1.13
10216	PRG4	proteoglycan 4	3	−1.11

*GEO database GSE40839, Lindahl et al. Respiratory Res 2013, 14∶80.

### Genes aberrantly methylated in IPF fibroblasts are enriched in annotation for various cell functions

**Figure 5:**
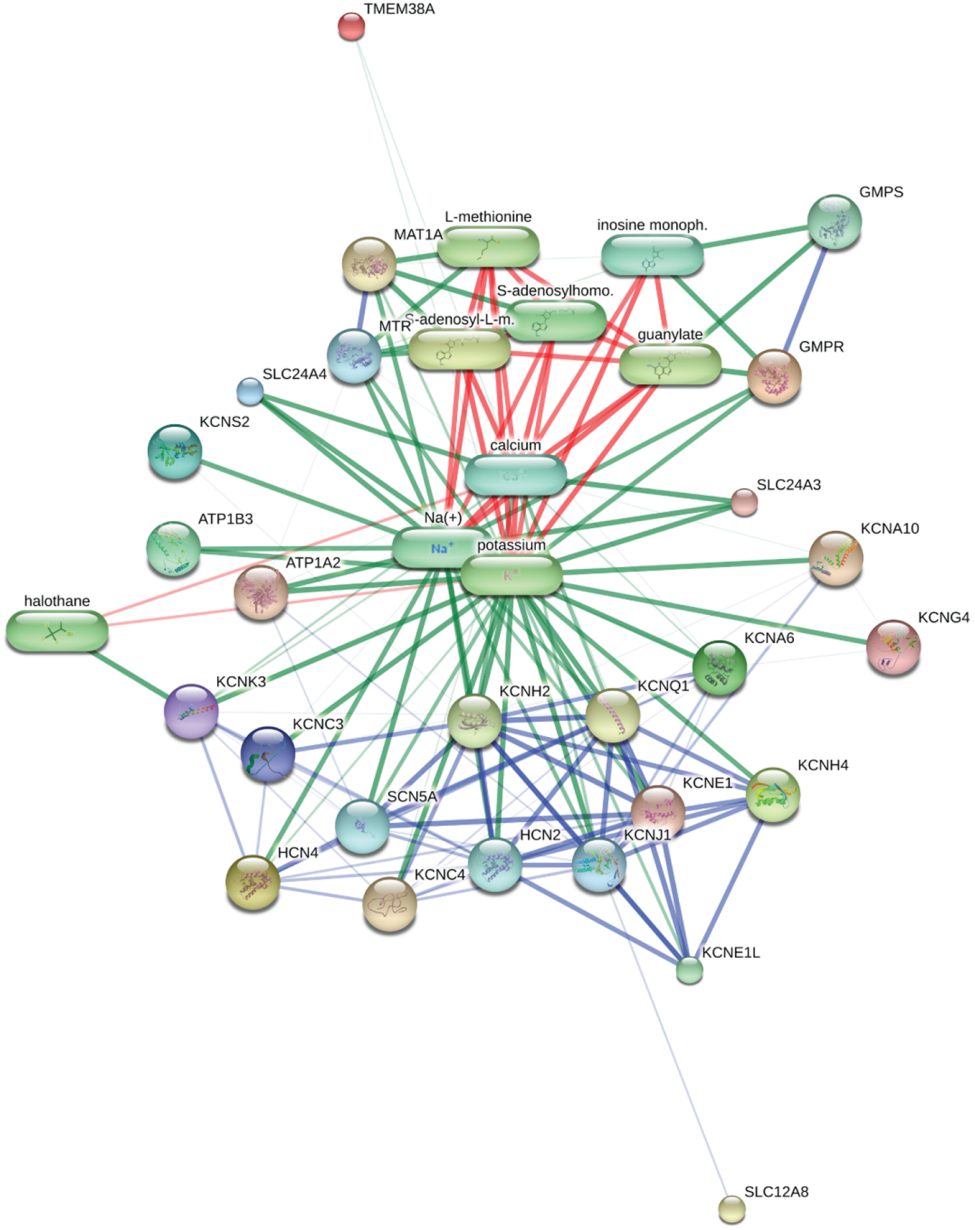
Network analysis of differentially methylated genes in potassium ion binding gene ontology (GO) concept. The GO category “potassium ion binding” was identified as an overrepresented concept in our dataset. The 18 differentially methylated genes with annotations in this category were analyzed by STITCH network analysis (http://stitch.embl.de) with their interrelationship shown. Protein-protein interactions are shown in blue, protein-chemical interactions are shown in green, and interactions between chemicals are shown in red.

To determine if the 732 differentially methylated genes we identified in IPF fibroblasts share common biological functions, we used ConceptGen (conceptgen.ncibi.org) [32] to identify “concepts” – categorized by gene ontology (GO) classifications, medical subject headings (MeSH), and other publicly available annotative data sets. Statistically significant enriched GO concepts are shown in Table 4 and include “extracellular matrix,” “organ morphogenesis,” and “potassium ion binding.” Other concepts enriched by MeSH and miR database annotations are listed in Table S5. Because “potassium ion binding” is an enriched concept that was unexpected in our analysis and has no obvious relationship to fibrogenesis, we performed a network analysis of genes in this concept to examine their interrelationship, which is shown in Figure 5.

**Table 4:** Enriched Gene Ontology Concepts Overrepresented in Annotation Among Genes Differentially Methylated in IPF.

Concept Name	Concept Type	Gene List Size	Overlap	P-Value	Q-Value	Overlaping Genes
extracellular region part	GO CellularComponent	771	67	2.75E-08	1.23E-05	CDH13,COL6A3,COL19A1,COMP,CRHBP,CSF3,APLP1,APOA1,BMP8B,C1QA,C2,C5,PI3,IL17A,LAMC2,LECT2,LGALS3BP,LGALS8, MMP3,MMP7,MMP17,MSMB,MATN2,OVGP1,HAS1,HPX,IGFBP2,FGF1,GDF2,MSTN,EREG, MFAP5,DLK1,PROM1,THPO,TIMP3,CRISP2,VTN,WNT6,WNT7B,WNT10B,CCL3,CCL8,CCL16,CXCL6,SOD3,SFTPB,SPP2,TAC1,IL1F5,PRG4, SCRG1,POSTN,CCL26,TNFSF13B,SPON2,SPON1,COL18A1,SMOC2,WFDC1,IL1F9,CYTL1,ADAMTS17,CMTM5,NTNG2,\IL1F10,ADAMTSL5
anatomical structuremorphogenesis	GO BiologicalProcess	841	64	2.25E-06	5.56E-03	ALDH1A3,CDH13,CDX4,CHRNA7,COMP,BCL2,APLP1,CA9,ATOH1,ADRA1D,PDGFRA,PPL,MID1,NEUROG1,ABLIM1,LOR,NGFB,NINJ2,MSX1,NOTCH4,KCNE1, NRTN,MAS1,MCAM,MYH7,SIX6,KNG1,KRT1,KRT4,GAMT,FGF1,FOXI1,EPB42,EREG,EYA4,DLG1,DLX5,GCM1,TBX4,ZNF22,PAX8,ULK1,SEMA3B, FOXN1,S100B,TNNI3,TWIST1,VEGFC,WNT7B,SOX2,SFTPB,SHB,CYFIP1,RHOQ,SPON2,COL18A1,ATP10A,DNAI2,ALX4,F11R,EGFL7,TEKT4,NTNG2,LHFPL5
extracellularspace	GO CellularComponent	509	46	2.62E-06	5.83E-04	CDH13,CRHBP,CSF3,APOA1,BMP8B,C1QA,C2,C5,IL17A,LECT2,LGALS3BP,LGALS8,MMP3,MMP7,MSMB,OVGP1,HPX,IGFBP2,FGF1,GDF2, MSTN,EREG,MFAP5,DLK1,PROM1,THPO,CRISP2,VTN,CCL3,CCL8,CCL16,CXCL6,SFTPB,SPP2,TAC1,IL1F5,PRG4,SCRG1,CCL26,TNFSF13B,SPON2,WFDC1,IL1F9,CYTL1,CMTM5,IL1F10
potassium ionbinding	GO MolecularFunction	123	18	1.57E-05	1.69E-02	ATP1A2,ATP1B3,KCNA6,KCNA10,KCNC3,KCNC4,KCNE1,KCNK3,MAT1A,KCNS2,GMPR, KCNH4,HCN4,TMEM38A,SLC24A3,SLC24A4,KCNG4, SLC12A8
organmorphogenesis	GO BiologicalProcess	359	33	3.18E-05	3.94E-02	ALDH1A3,CDH13,CDX4,CHRNA7,COMP,APLP1,ATOH1,PDGFRA,NEUROG1,ABLIM1,MSX1,NOTCH4,MYH7,SIX6,KRT1,GAMT,FGF1,FOXI1,EREG,DLX5,TBX4,ZNF22,FOXN1,TNNI3,VEGFC,WNT7B,SOX2,SFTPB,SHB,COL18A1,ALX4,EGFL7,LHFPL5
extracellularmatrix	GO CellularComponent	305	28	2.85E-04	4.13E-02	COL6A3,COL19A1,COMP,APLP1,PI3,LAMC2,LGALS3BP,MMP3,MMP7,MMP17,MATN2,HAS1,MFAP5,TIMP3, VTN,WNT6,WNT7B,WNT10B,SOD3,SFTPB,POSTN,SPON2,SPON1,COL18A1,SMOC2,ADAMTS17,NTNG2,ADAMTSL5

### IPF fibroblasts exhibit heterogeneity in their methylation profiles

**Figure 6:**
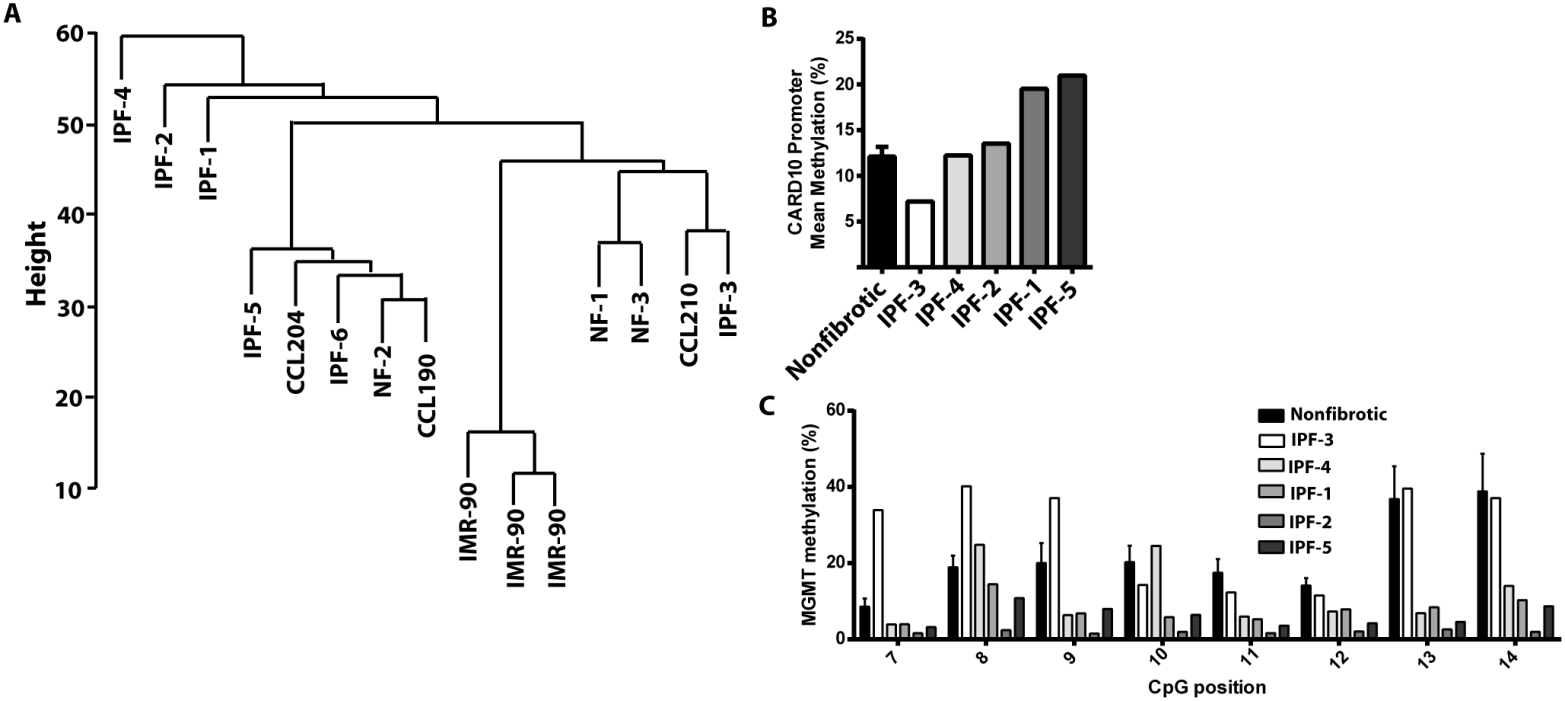
Variability in DNA methylation of IPF cells. A) Heirarchical cluster analysis was performed in each cell line studied, which also includes 3 separate samples of IMR-90 cells, a primary fetal fibroblast cell line. The mean methylation levels of the upstream *CARD10* promoter (B) and the methylation levels of the individual CpG sites in the *MGMT* promoter (C) were compared among each individual IPF cell line and nonfibrotic cell lines.

**Figure 7:**
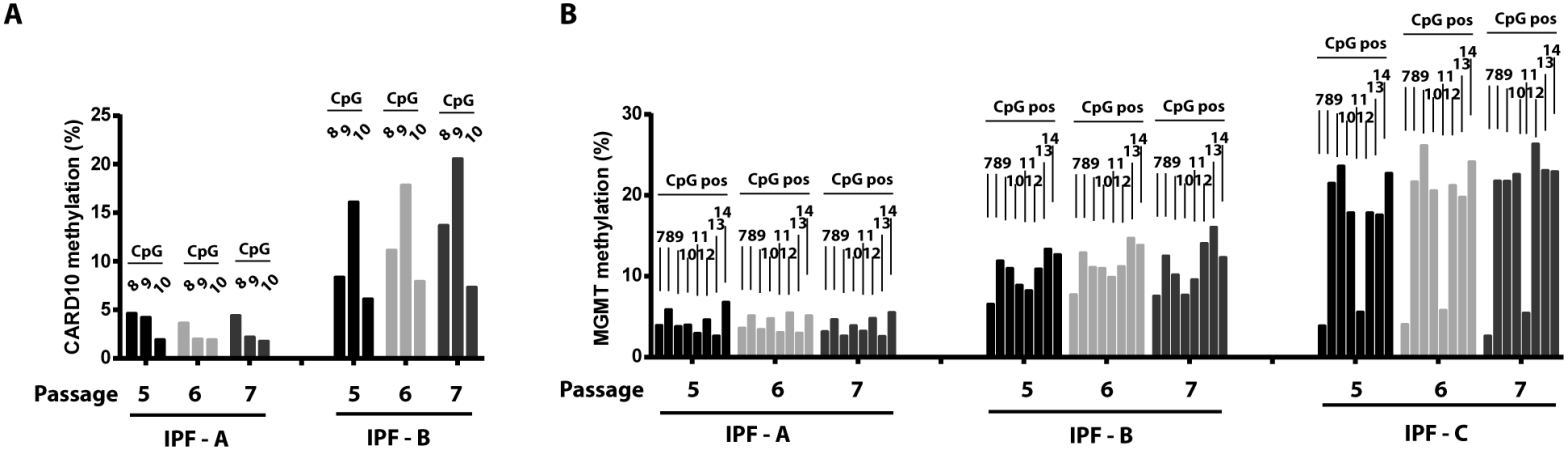
Stability of DNA methylation differences with cell passage. Three different IPF cell lines (A, B, and C) were assessed at passages 5, 6, and 7, and the DNA methylation for each cell line and each passage was compared. A) Shown are the methylation level of CpG sites 8, 9, and 10 in the upstream segment of the *CARD10* promoter for IPF cell lines A and B at serial passage. B) Shown are the methylation levels of CpG sites 7–14 of the *MGMT* promoter for IPF cell lines A–C at serial passage.

Although we identified significant differences in mean DNA methylation between IPF and nonfibrotic control cells, considerable variability in methylation was present among individual IPF cell lines. Hierarchical cluster analysis identified a group of IPF lines that exhibited similar overall DNA methylation profiles, with other lines exhibiting divergent profiles (Figure 6A). This variability was evident even when the mean methylation of an individual gene, such as *CARD10* was compared among IPF lines (Figure 6B). Culture conditions have been suggested to influence global DNA methylation levels [33], [34] and one might speculate whether *in vitro* culturing of fibroblasts is a source of variability in DNA methylation. All cell lines were analyzed at similar passage during our analysis. However, we found substantial consistency in the DNA methylation levels of *CARD10* and *MGMT* even when cells were examined at different passage numbers (Fig. 7), consistent with the heritable nature of DNA methylation levels.

## Discussion

Excessive fibroproliferation is a key hallmark of IPF, and fibroblasts are the major effector cell responsible for this process [35]. Work from numerous laboratories has demonstrated that fibroblasts from IPF patients exhibit an abnormally “activated” phenotype [3], [5]–[7], which in turn has been attributed to differential regulation of key genes [3], [4], [7], [8], [10], [12]–[14]. These differences in gene expression typically persist over time, through cell division, and despite changes in their microenvironment. Epigenetic mechanisms represent an attractive explanation for such stable differences in gene expression, but global methylation analysis of IPF fibroblasts has not previously been reported. Here, we used the HumanMethylation27 Bead-Chip array to compare the global DNA methylation patterns of IPF and nonfibrotic control fibroblasts. We identified multiple CpG loci and their associated genes that are differentially methylated; these methylation differences spanned the genome, were present in every chromosome, and were associated with differences in gene expression. Some of these differentially methylated genes have known effects on fibroblast biology, and their dysregulated expression can readily be envisioned to contribute to the pathogenesis of IPF. Other genes possessed annotations enriched in gene ontology classes such as potassium ion channels and organ morphogenesis that we found on first glance to be surprising. However, literature review of some of these genes in other organ systems suggests that these too, may be important to fibrosis. Additional studies will be needed to determine if these represent previously unappreciated novel pathways involved in IPF pathogenesis. Taken together, our findings establish DNA methylation as a critical epigenetic mechanism that contributes to the altered phenotype of IPF fibroblasts.

Although alterations in DNA methylation contribute to the pathogenesis of many diseases, the importance of this epigenetic modification in IPF is only beginning to be realized. Two independent studies have shown that tissue from the lungs of IPF patients exhibit global DNA methylation differences compared to nonfibrotic lungs [15], [16]. However, numerous cell types comprise the lung parenchyma, and mixtures of different cell types present in the whole lung tissue utilized in these studies might have obscured important DNA methylation differences present in any given cell. This is especially problematic considering that certain cell types, such as epithelial cells and fibroblasts, are known to exhibit, dichotomous gene expression profiles. It is therefore not surprising that many of the methylation differences identified by these studies of whole lung were not observed in our studies of lung fibroblasts, despite use of the same array platform (i.e. HumanMethylation27 array) [16]. Studies of specific genes – namely, Thy-1 [19], *PTGER2* [18], and p14^ARF^ [17] – have identified their differential methylation in IPF fibroblasts, but this is the first study of which we are aware that describes genome-wide differences in DNA methylation in these critical mesenchymal effector cells.

Bisulfite sequencing allowed us to validate the differential methylation of *CDKN2B*, *CARD10*, and *MGMT*, and we found that differences in methylation of these genes involved CpG sites not represented by the array. These genes exemplify both differential hypermethylation (in the case of *CDKN2B* and *CARD10*) and hypomethylation (in the case of *MGMT*), contributing to their differential gene expression in IPF fibroblasts. CDKN2B is an endogenous cell cycle inhibitor that binds to CDK4 and -6 [30]; its decreased expression in IPF fibroblasts may contribute to their increased proliferation. Indeed, silencing of CDKN2B in normal fibroblasts resulted in increased cell proliferation. CARD10 is a scaffold protein recognized to associate upstream G protein signals (such as that from the putative fibrogenic lipid mediator lysophosphatidic acid [LPA]) with downstream NF-κB activity [36], [37]. MGMT is a DNA repair enzyme that regulates chromatin stability and susceptibility to apoptosis [38] and its increased expression in IPF cells may contribute to the well-recognized phenomenon of fibroblast resistance to apoptosis in IPF [3], [9], [10], [12]. Further studies at the molecular and biochemical levels are needed to characterize the biological significance of each of these genes, as well as others identified in the array, in IPF. We compared our set of differentially methylated CpG loci with publicly available array data that compared gene expression between IPF and normal fibroblasts, and noted that 52 of the reported differentially expressed genes [31] overlapped with our dataset. This was not a result of mere chance, as the false discovery rate was 7.23×10^−5^. However, we also note that such *in silico* analysis has limitations due to the different platforms used, and variability in patient population between institutions and cell isolation techniques between different laboratories. Nonetheless, this suggests that some of the differences in gene expression of IPF fibroblasts may be attributable to differences in DNA methylation. It is noted that not all of the differences in gene expression were directionally opposite to the differences in methylation, suggesting that further studies would be needed to determine how differences in individual gene expression may be affected by either hyper- or hypomethylation. Although the patient selection criteria, biopsy techniques, and culture methods employed by Lindahl et al. were very similar to those utilized in our study, future experiments where DNA methylation and gene expression analysis are performed from identical samples in parallel may improve the robustness of matching DNA methylation and gene expression data.

Enrichment analyses revealed that certain genes that were differentially methylated were overrepresented in annotation for gene ontology classes such as “extracellular matrix” and “extracellular space.” The enrichment of these concepts is consistent with a disorder that is characterized by tissue remodeling due to excess deposition of matrix proteins such as collagens. Still, identifying the genes within these gene ontology classes that are differentially methylated may provide insight into the importance of each particular gene in driving fibrosis. The fact that these particular gene ontology classes were found overrepresented in annotation also validates the biological significance of the larger array findings and indicates that observed methylation differences occur in genes that are functionally relevant to IPF pathogenesis. We also identified unexpected gene ontology concepts such as “organ morphogenesis” and “potassium ion binding” that were enriched in our gene data set. Some genes within the “organ morphogenesis” gene ontology, such as *PDGFRA*, *TWIST1*, *WNT7B*, and *SFPTB* [39]–[42], have been implicated in IPF pathogenesis; other genes in the “potassium ion binding” gene ontology have been implicated in renal fibrosis [43] and further interrogation of the function of these genes could drive the discovery of novel mechanisms of IPF pathogenesis. Network analysis of these genes reveals that they share closely coordinated functions, based on their direct protein-protein interaction and on their ability to bind potassium ion and serve as a potassium ion channel. Future work may reveal a novel role for potassium signaling in IPF pathogenesis.

Our study had important limitations. Because parenchymal lung fibroblasts are typically obtained from surgical lung biopsies and because this invasive procedure is infrequently performed in IPF and control patients, we were limited to a relatively small sample size. The presence of variability among samples might have further obscured important methylation differences that might be identified if a larger sample size was used, and indeed initial statistical analysis with a more stringent false-discovery rate revealed no significant differences. This prompted us to take a less stringent, “fold change-ranking with a non-stringent p-value cutoff” approach, a statistical paradigm that has been validated in other expression [25]–[27] and DNA methylation microarray studies [24]. Most importantly, however, independent analysis of the DNA methylation, gene expression, and function of individual genes such as *CDKN2B*, *CARD10*, and *MGMT* indicate that this less-stringent approach still allows us to identify true methylation differences that are biologically important in IPF fibroblasts. We were also able to link methylation differences in genes with differential gene expression patterns from an independent data set generated by other investigators [31]. Finally, we compared IPF fibroblasts to two sets of nonfibrotic controls – those from commercial cell lines and those from histologically normal regions of lung in patients who underwent resection for lung nodules. Although comparing IPF fibroblasts to commercial cell lines circumvents the potential of lung cancer to exert a “field effect” [21], [22] on the DNA methylation of surrounding fibroblasts, commercial lines are derived from younger subjects that are not appropriately age-matched compared to patient-derived controls. This difference in age may bias results, since DNA methylation may change with age [44]. To mitigate against these potential limitations and biases, we compared IPF cells with two different control groups, and suggest that this allowed us to generate a more robust list of differentially methylated genes specific to IPF.

We also recognize that the IPF fibroblasts in our study were predominantly from female patients while our normal control fibroblasts were mostly from male subjects. This was coincidental due to the small sample size as IPF is slightly more common in men [45], [46], but differences in gender could be a confounding factor in our study as gender has been demonstrated to impact DNA methylation levels, even independent of X chromosomal differences [47], [48]. We do note that none of the top 50 differentially methylated genes (Table S1) between IPF and nonfibrotic control cells are on sex chromosomes, and of the 787 total differentially methylated CpG loci, only 21 of them are on the X chromosome. However, differences in gender and age are potentially confounding variables that could influence our findings of DNA methylation differences, and have to be considered in future methylomic analyses and in follow-up studies of individual CpG loci.

Up to 3,690 of 27,578 CpG loci (13.4%) on the HumanMethylation27 array are annotated for known SNPs. The inclusion of probes with SNPs has the potential to be problematic, as differences in signals from these probes may be falsely attributed to methylation differences when in fact, they are due to SNP variations in the samples. However, since the proportion of CpG loci (∼14%) that we identified as differentially methylated and annotated for SNPs is similar to the proportion of SNPs annotated over the entire array, the data do not suggest that SNPs represent a bias in the identification of differentially methylated CpG sites. Although probes with annotated SNPs could have been removed in the analysis, this has the potential for creating its own significant (and, to date, unknown) bias. The frequency of some minor SNP alleles may be quite low, such as 1% or even 0.1%, and removing these data points because they reside on SNPs of low frequency might not be justified. Furthermore, we confirmed differential methylation in the promoters of three genes (*CDKN2B*, *CARD10*, and *MGMT*) by bisulfite sequencing. Of these three, both *CDKN2B* and *MGMT* are annotated for known SNPs in the relevant sites, but both are validated as being differentially methylated. Finally, the presence of SNPs and their ability to affect DNA methylation levels might actually be important biologically. Thus, we did not *a priori* eliminate probes with known SNPs in our analysis, but instead annotated them in Tables 2, S2, S3, and S4. These annotated loci should not be ignored, but considered with caution in future follow-up studies.

IPF fibroblasts exhibited significant heterogeneity in their global DNA methylation patterns, as evident in hierarchical clustering analysis and in the DNA methylation analysis of individual genes. As compared to other fibrotic lung disorders, IPF is recognized to be clinically heterogeneous [49], and to exhibit pathologic heterogeneity (defined by areas of seemingly normal histology adjacent to areas of dense fibrosis) that distinguishes it from other types of interstitial lung disease [1], [50]. One might thus speculate that even the process of culturing cells *in vitro* may produce populations of cells with distinct patterns of DNA methylation. Despite this, we were able to identify many differences in DNA methylation even among a small sample size. Systematic comparison of methylation levels of *CARD10* and *MGMT* genes between different passages confirmed that DNA methylation patterns are stable through cell division. This is a central, albeit not often tested, tenet of epigenetics. It is presumed that heterogeneity could be reduced if a larger sample number was used or if specific subpopulations of fibroblasts, segregated by patient clusters or by the region of lung from which cells were isolated, were employed. In fact, methylation differences in *THY1* [19], *PTGER2* [18], and *p14^ARF^* [17] were all previously shown to be heterogeneous among IPF fibroblasts, which may account for why these genes did not show up in our array analysis. This suggests that even cells of the same cell type, obtained from geographically similar regions of the lung, may exhibit heterogeneity in DNA methylation and gene expression. Heterogeneity also suggests that examining mean methylation differences between IPF and nonfibrotic control cells may obscure important differences that may only be evident if subpopulation of IPF samples, defined *a priori* by unsupervised hierarchical clustering of IPF patients, were performed. Despite the variability, we were still able to identify certain CpG loci that differed in methylation between IPF and nonfibrotic fibroblasts as a whole. Finally,the array assays on average only 1–2 CpG sites per gene and is biased towards sites within CpG islands. Future work with arrays that assay 450,000 CpG sites and next-generation sequencing approaches that provide greater unbiased coverage may reveal many more differentially methylated CpG loci that eluded our detection.

How DNA methylation differences arise in IPF is unknown. We previously reported that the antifibrotic mediator prostaglandin E_2_ increases DNMT3a expression [28], and that expression of different DNA methyltransferases (DNMTs) vary in IPF fibroblasts [18]. However, alterations in DNMT expression is likely to only partially explain all of the DNA methylation differences observed in IPF cells, and does not account for how certain genomic regions are specifically targeted. How DNA methylation differences affect gene expression on a gene-by-gene basis is also unclear, especially since our data suggest DNA methylation differences in IPF are frequently found in non-CpG islands, and when compared to expression array data from other investigators, show varying levels of correlation between gene expression and methylation of specific CpG loci. Despite these unresolved issues, our data provide the first description of the global DNA methylation differences that are present in IPF fibroblasts, and offers a starting point for understanding the extent of such differences, and the importance of the genes affected by DNA methylation in IPF pathogenesis.

## Supporting Information

Table S1 **Primers for Pyrosequencing.**
(XLSX)

Table S2 **Differentially methylated CpG sites in IPF versus patient-derived nonfibrotic fibroblasts.**
(XLSX)

Table S3 **Differentially methylated CpG sites in IPF fibroblasts versus normal commercial line fibroblasts.**
(XLSX)

Table S4 **Differentially Methylated CpG Sites in Common When IPF Cells Were Compared to Both Control Groups.**
(XLSX)

Table S5 **Enriched Concepts Overrepresented in Annotation Among Genes Differentially Methylated in IPF vs Nonfibrotic Control Cells.**
(XLSX)

Table S6 **Enriched Concepts From Genes Hyper- or Hypomethylated in IPF Fibroblasts Compared to Nonfibrotic Control Cells.**
(XLSX)
